# Risk Analysis and Assessment of Lipid Abnormalities as the Earliest Complication in Newly Diagnosed Diabetic and Non-Diabetic Individuals of a Local Population

**DOI:** 10.3390/healthcare10112308

**Published:** 2022-11-18

**Authors:** Zunaira Ali Baig, Amir Rashid, Asifa Majeed, Zahra Masood, Asma Faryal, Zahra Arshad Khan, Aden Razaq

**Affiliations:** Department of Biochemistry and Molecular Biology, Army Medical College, National University of Medical Sciences, Rawalpindi 46000, Pakistan

**Keywords:** diabetes mellitus, dyslipidemia, hypertriglyceridemia, lipoprotein

## Abstract

Lipid variations have been frequently observed in global populations that can affect health status. Mainly studies have been conducted on the type 2 diabetic population, but limited data is available on newly diagnosed ones to unravel complications and risk predictors independent of disease progression. This study comprising 244 individuals was carried out to assess the lipid abnormalities in newly diagnosed diabetics and non-diabetics. The clinical and socio-demographic data were collected and analyzed using independent samples t-test and linear regression. Serum lipid variations were observed individually and in combination. The individuals in group I (diabetics with dyslipidemia) revealed elevated levels of low-density lipoprotein and serum triglycerides higher than in group II (non-diabetics with dyslipidemia). The frequency of deranged total cholesterol in group I was observed to be higher than in group II. Independent samples t-test showed a significant mean difference in variables between the two groups. Linear regression analysis showed a significant variable outcome for predictors between high-density lipoprotein (HDL) and physical activity (B= −0.043, 95% CI: −0.80, −0.006) and total cholesterol (TC) with family history (B= −0.062, 95% CI: −0.123, −0.001). The findings conclude that lipid levels deranged independently regardless of type 2 diabetes mellitus and present as an early onset in type 2 diabetes instead of later stage complication. These derangements of lipid levels are an independent risk factor for future cardiovascular pathology.

## 1. Introduction

Non-communicable diseases are expanding at a larger scale, and their global mortality rate is 71%. Various factors are involved in disease development ranging from environmental to genetic [1]. Variations in the lipid parameters such as total cholesterol (TC), high-density lipoprotein cholesterol (HDL-C), low-density lipoprotein cholesterol (LDL-C), and triglycerides (TGs) mark the development of dyslipidemia. Lipid abnormalities constitute the risk factor for cardiovascular diseases (CVD). The global prevalence of various metabolic disorders, including dyslipidemia, is continuously rising at an alarming rate. Different prevalence patterns of lipid abnormalities have been reported in various countries [2,3,4,5]. Uncontrolled hyperglycemic conditions also contribute to the alteration in lipid metabolism, thus posing a great risk for developing cardiovascular events. Dyslipidemia (hypertriglyceridemia being at highest—61.9%) was observed to be higher among the Arabs with poorly controlled glycemic status [6]; similar findings were observed in the Pakistani population [7]. About 63% of the local subjects had shown lipid derangements. Among the varying lipid parameters, low HDL-C was observed to be frequent (17.3%) [8]. The high frequency of varying lipid parameters presents an independent risk factor for cardiovascular pathologies. Various epidemiological studies have shown a strong association between lipid variations with cardiovascular disease risk. Hypercholesterolemia and other lipid derangements have been established as significant risk factors for cardiac events and stroke [9,10]. According to WHO reports, this outcome can cause 2.6 million deaths per year globally [11].

Several factors can cause lipid derangements, including diabetes (T2DM), hypertension, obesity [12], and family history [13]. Dietary patterns, lifestyle, and various metabolic disorders can also constitute the onset of dyslipidemia. Insulin resistance has an impact on lipid metabolism modification. Generally, clinicians consider dyslipidemia as a late-onset complication of diabetes [14,15,16,17]. A local study showed the presence of disturbed lipid profiles in newly diagnosed as well as known diabetics [18]. Dyslipidemia alone and in diabetes is a risk predictor for the progression of CVDs. The growing number of individuals with deranged lipids is a matter of health concern. The earlier screening of lipid abnormalities in populations with and without hyperglycemic status might serve as an effective health management strategy. This can be achieved with the timely identification of lipid variations and the effective execution of lifestyle modification.

Limited studies are available regarding newly diagnosed type 2 diabetics in our population. Deranged lipid parameters can be present in individuals independent of hyperglycemic status, influenced by various factors. Therefore, this study aimed to identify and assess the lipid abnormalities and evaluate risk predictors for developing dyslipidemia in the newly diagnosed type 2 diabetic and non-diabetic adult populations. This study will help establish future screening programs to identify lipid abnormalities in the general population.

## 2. Methods

### 2.1. Study Design and Setting

This cross-sectional study was carried out at the Department of Biochemistry and Molecular Biology of Army Medical College, National University of Medical Sciences Rawalpindi, Pakistan, from 10 November 2020 to 8 February 2021 in collaboration with the clinical OPD of the Endocrinology Department of Pak Emirates Military Hospital Rawalpindi, Pakistan. The study was conducted in compliance with the principles of the Declaration of Helsinki and carried out after the formal approval of the Institutional Ethical Review Committee (ERC Ref dated 2 April 2019).

### 2.2. Subjects and Data Retrieval

Participants recommended for fasting lipid profiles, and blood sugar levels were recruited using non-probability convenience sampling. Newly diagnosed diabetics and non-diabetics were enrolled with lipid derangements, and subjects with comorbidities were excluded from the study. After taking their informed written consent with their signatures or thumbprints, their demographical data were recorded in a designed proforma. All the participants’ data were kept confidential. Their clinical parameters record was then obtained from the hospital’s pathology laboratory. The study comprised 244 participants, who were further grouped into diabetics and non-diabetics based on their lipid profile as group I (diabetics with dyslipidemia) and group II (non-diabetics with dyslipidemia). Plasma glucose ≥ 7.0 mmol/L was regarded to be diabetic, while dyslipidemia was considered if one of the following lipid parameters were present exceeding their cut-off values: TC ≥ 5.2 mmol/L, TG ≥ 1.70, HDL-C < 1.0 mmol/L and LDL-C ≥ 2.59 mmol/L [19]. The data were collected on socioeconomic status, physical activity, and family history, along with lipid profile and blood sugar level in fasting.

### 2.3. Statistical Analyses

The data were statistically analyzed using the IBM SPSS (statistical package for social sciences) software version 22.0 (IBM Corporation, New York, NY, USA). Data are presented as percentages and means ± SD. An independent sample t-test was employed to compare the mean difference of variables between the groups. Linear regression was used to assess the relationship between lipid parameters and demographic factors. A *p*-value ≤ 0.05 was considered statistically significant for all parameters.

## 3. Results

### 3.1. Clinical Analyses of Subjects

**Figure 1:**
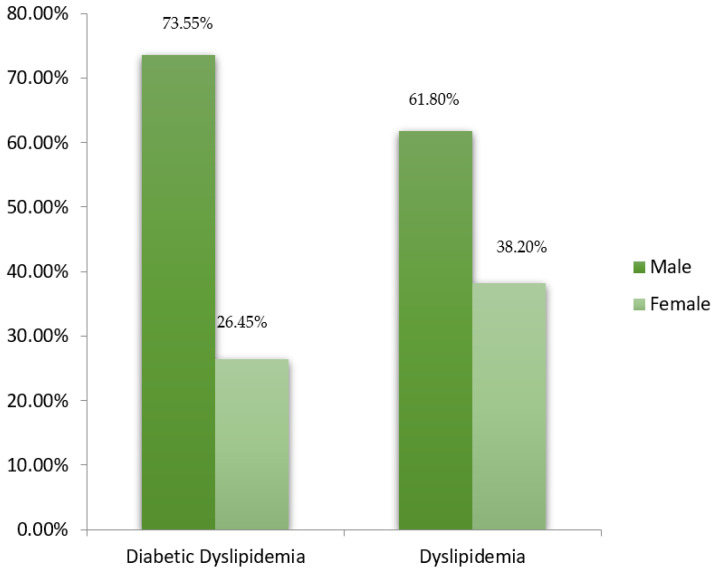
A bar graph illustrating the distribution of stratified males and females in the categorized groups.

A total of 244 participants were included in the study, out of which 169 (69.3%) were males, and 75 (30.7%) were females. They were further categorized into two groups based on their pattern of lipid and fasting blood sugar profiles. There were 155 (63.52%) subjects in the diabetic dyslipidemia group, while non-diabetics with dyslipidemia group had 89 (36.48%) study participants. The mean age for the female study participants was (46.95 ± 12.25 years), and male study participants had a mean age of (51.89 ± 11.21 years). Within the groups, 155 (63.5%) participants were with diabetic dyslipidemia, and 89 (36.5%) were dyslipidemic stratified into males and females within the groups (Figure 1).

Serum lipid alterations were observed individually and in combination with diabetes. Among the individuals with diabetic dyslipidemia, 47 (30.32%) had combined lipid abnormalities, while dyslipidemic individuals were presented to be 11 (12.36%) with all deranged lipid parameters, i.e., TC, TG, HDL-C, and LDL-C. Individually, diabetic dyslipidemia and the dyslipidemic groups had 77 (49.68%) and 24 (26.97%) subjects with deranged TC. The frequency of elevated LDL-C was 73.55% in group I and 64.04% in group II in reference to the cut-off value. Serum TG levels more than the cut-off values were observed to be 86.45% and 75.53% in individuals of groups I (*n* = 155) and II (*n* = 89), respectively. Similarly, 138 (89.03%) dyslipidemic individuals with diabetes presented deranged HDL-C values, while 76 dyslipidemic individuals (85.39%) were observed to have deranged HDL-C. Total cholesterol was observed in 72 (46.45%) diabetics with dyslipidemia, while 24 (26.97%) in non-diabetics with dyslipidemia.

Both the study groups exhibited a comparatively higher proportion of reduced high-density lipoprotein and deranged TGs than any other lipid parameter. Subjects from groups I and II showed higher levels of TGs second to HDL, LDL second to HDL, and TC as the least high in proportion.

### 3.2. Demographic Analyses of Subjects

**Figure 2:**
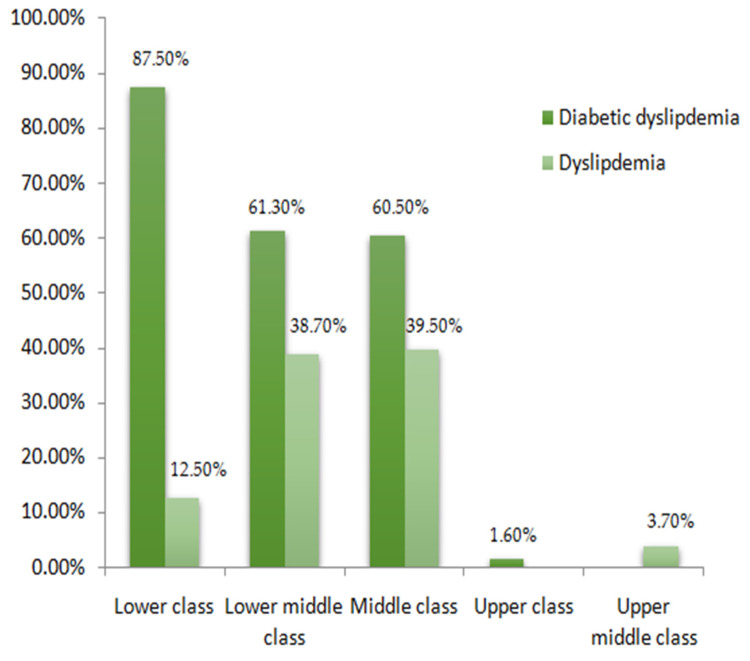
Socio-economic status of group subjects.

**Figure 3:**
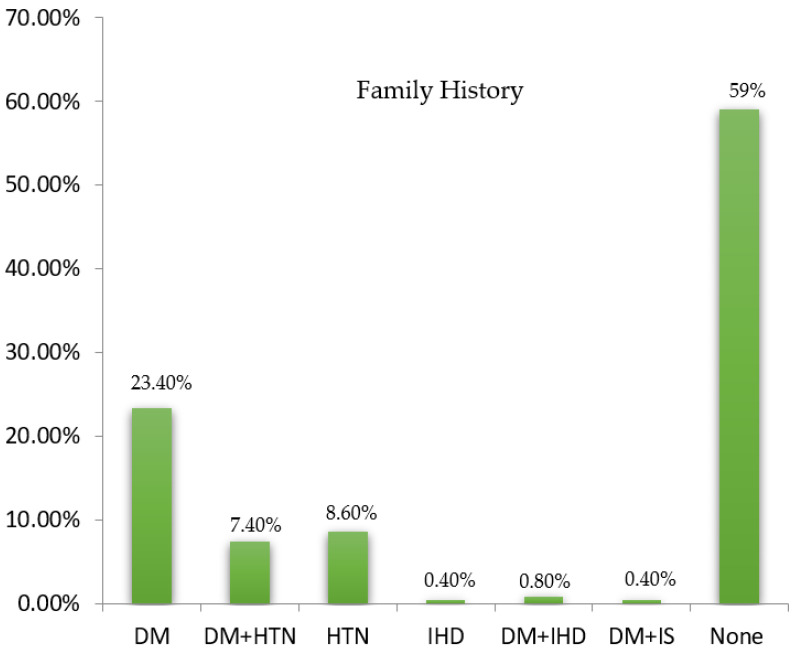
A bar chart illustrating the proportion of subjects with family history.

**Figure 4:**
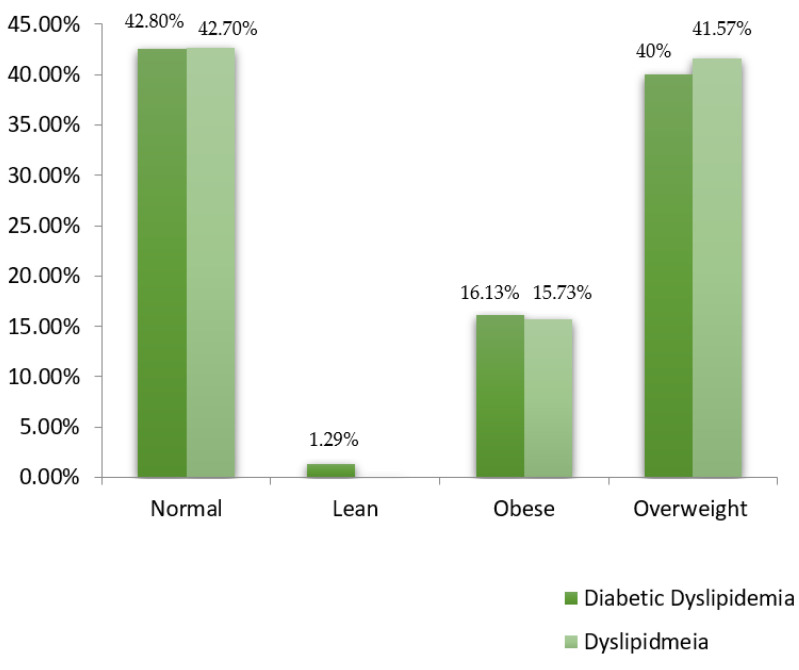
A bar chart representing the proportion of study subjects with reference to their BMI.

The socioeconomic status of subjects was constructed by determining their social status and occupation and classified into low, lower middle, middle, upper middle, and upper classes. Considering the socioeconomic status of the included study subjects (*n* = 244), 40 (16.4%) belonged to the lower class, 62 (25.4%) were from the lower middle class, and 129 (52.87%) were from the middle class. Only four (1.64%) belonged to the upper class comprising subjects from diabetic dyslipidemia, and nine (3.7%) belonged to the upper-middle class with subjects from the dyslipidemic group. Figure 2 presents the proportion of individuals stratified in both groups with socioeconomic status. Regarding the family history, 57 (23.36%) had a family history of T2DM, 18 (7.38%) subjects had diabetes mellitus and hypertension, two (0.82%) had diabetes mellitus and ischemic heart disease, while only one (0.4%) individual had diabetes mellitus and ischemic stroke, 21 (8.6%) were found to have a family history of hypertension, one (0.4%) had ischemic heart disease alone, while 144 (59%) had no family history of any disease. More individuals were observed to have diabetes mellitus alone among disease family history. The proportion of the family history among the groups’ subjects is illustrated in a pie chart (Figure 3). The physical activity of the study participants was determined through a time duration questionnaire each individual spent on exercise and home chores. Physical activity was classified into active, moderate, and inactive. Concerning the physical activity among the study subjects, 126 (51.64%) were moderate, 36 (14.75%) had an active status of physical activity, 62 (25.41%) were inactive, while 20 (8.2%) individuals did not show any activity response. The diabetic dyslipidemia group had 66 normal, 2 lean, 25 obese, and 62 overweight subjects, while the dyslipidemia group had 38 normal, 14 obese, and 37 overweight subjects (Figure 4).

### 3.3. Statistical Analyses

Independent samples *t*-test was used to determine the mean difference of continuous variables between groups. The mean difference between groups in age, fasting blood sugar, HDL, TC, TGs, and HbA1c was observed to be significant (*p* ≤ 0.05) (Table 1). The linear regression was employed between lipid parameters and predictors, including body mass index (BMI), socioeconomic status, physical activity, and family history. The analysis revealed physical activity as a significant predictor for low HDL (*p* = 0.022, *p* < 0.05), gender as a predictor for abnormal TGs (*p =* 0.014, *p* < 0.05), family history for deranged TC (*p* = 0.045, *p* < 0.05) in the groups (Table 2). R squared shows the variability of the dependent variable with its predictor outcome. HDL shows 3.4% variability in the response data. A 3.9% and 4% variability was observed for TGs and TCs, respectively. The low R squared value reveals that much of the variation in data could not be explained, but significance in the observed response predictors shows some influence.

## 4. Discussion

Dyslipidemia is a global health concern and is considered a key determinant of developing cardiovascular disease. Developing countries have observed higher lipid derangements which serve as a crucial health challenge. Most studies on lipid abnormalities have been conducted in diagnosed diabetic individuals but minimal in newly diagnosed ones. Thus, this study assessed the pattern of lipid derangements in newly diagnosed diabetics and non-diabetics. The findings bring to highlight the need to control diabetes and dyslipidemia globally. The findings may provide a guideline for healthcare policymakers to devise better healthcare plans to reduce national resources’ overburden. This will add to the pool of knowledge of health practitioners to get insight into dyslipidemia independently to circumvent the future risk of CVD and improve the well-being of the community.

Abnormal serum lipid levels have been well-established to have a strong association with various heart conditions. Deranged lipid parameters have been observed to increase the risk of myocardial infarction [20,21]. Dietary patterns and a sedentary lifestyle constitute factors for diabetes and dyslipidemia in our population. Obesity [22] and diabetes have been well studied in modifying the lipoprotein composition and thus count for developing dyslipidemia [23]. Clinicians usually describe dyslipidemia as a late complication of diabetes, but our study data shows the presence of lipid abnormalities in newly diagnosed diabetic subjects. This demonstrates the onset of dyslipidemia as an independent risk factor. In comparison, the data from various local studies revealed diabetes mellitus as a predictor of dyslipidemia onset [24,25].

The current study has analyzed the spectrum of abnormal lipid profiles in individuals with and without hyperglycemia. The frequency of altered lipid profiles consisting of reduced HDL and hypertriglyceridemia was more prevalent among other lipid abnormalities in both groups. In various global populations, abnormal HDL and TGs have been widely observed as the most frequent lipid abnormality. This is in line with the findings of another national study presenting abnormal TGs and HDL-C (80.4% and 64%, respectively), with HDL being a moderate risk for coronary heart disease [13].

In a Chinese population, dyslipidemia was observed to be 48.27% [5] which is contrary to our study (36.48%) for individuals (group II) experiencing abnormal lipid profiles. The hyperglycemic status also contributes to changing lipid metabolism. Our study reports a slightly higher number of dyslipidemic individuals (63.52%) with hyperglycemic status (newly diagnosed diabetics) out of two lipid abnormality groups (group I and II) which is similar to the findings of a study in China [26].

Overall, our study presented a higher occurrence of dyslipidemia with and without diabetes in males (73.55% group I, 61.8% group II) than in females. Comparable findings were observed in international studies with varying frequencies. In Turkish subjects, more females (80.4%) were observed to have lipid abnormalities as compared to males (78.7%) [27]. In comparison with our study findings, a South African-based study observed a higher frequency of dyslipidemic females (75.79%) as compared to males (24.2%) [28]. Similar to previous local studies, patients having uncontrolled HbA1c revealed more lipid abnormalities [7].

Our study shows a higher proportion of reduced HDL in both the included group participants as compared to the findings of the study, revealing higher TGs (58%) in subjects with type 2 diabetes [29]. Contrary to our findings, another study demonstrated high TC (51.2%) as compared to HDL (28.2%) and TGs (27.6%) among the subjects [30]. Diet and lifestyle play a key role in affecting health status. Our investigations showed a higher proportion of sedentary lifestyles among female participants (42.67%) than in male participants (27.81%), which is in contrast to the study, which revealed unhealthy eating habits and sedentary lifestyles to be higher in males than females, contributing to the risk of dyslipidemia and hypertension [31]. In accordance with the findings of a study [32], this study reports more females (26.67%) as inactive as compared to males (21.30%) with varying lipid parameters. Data from the studies propose high levels of TGs and decreasing HDL-C count for the onset of coronary heart diseases [33,34]. In our study, varied serum levels of TGs and HDL-C out of deranged lipid parameters were found in a higher proportion for both the study groups. While the serum TG value with a more deranged form (≥5.00 mmol/L) was observed more (10%) in newly diagnosed diabetic subjects with dyslipidemia than in only dyslipidemic patients (7%). Similarly, serum HDL-C value in extreme deranged form (≤0.5 mmol/L) was found to be higher in newly diagnosed diabetic study subjects with modified lipid profiles (14.23%) as compared to dyslipidemic subjects (9%). In accordance with our observations, a local study revealed isolated high TG and low HDL-C as a more existing pattern of dyslipidemia [8]. HDL-C is a main component of reverse cholesterol transport and clears excess cholesterol from the body. Therefore, reduced HDL-C indicates a possible risk factor for the development of future CVD and is also linked with abnormalities of other lipoproteins. There are several factors influencing the levels of lipoproteins in the human body, including diabetes, obesity, a sedentary lifestyle, environment, and genetic predisposition. Our study marked dyslipidemia as an independent risk factor for developing cardiac events, as the participants were recruited with newly diagnosed diabetic status. Linear regression analysis was applied between lipid and demographic parameters to determine the risk predictor. There was no direct association found between lipid parameters and independent variables; however, among the predictors, physical activity was found to be a significant predictor for the deranged HDL (*p* = 0.022, *p* < 0.05), gender as a predictor for abnormal TGs (*p* = 0.013, *p* < 0.05), family history for deranged TC (*p* = 0.045, *p* < 0.05) in the groups.

In accordance with our observations, findings from a study depicted HDL as the most common lipid abnormality as well as physical activity status was observed to be the crucial determinant of the development of dyslipidemia [35]. However, in contrast to our outcome, a strong direct correlation of HbA1c was observed between reduced HDL levels and elevated BMI among the subjects of diabetic dyslipidemia [36]. Obesity has been observed to cause serum lipoprotein variation [37], and our study shows that a favorable HDL profile is associated with a physically active status.

Our study had some weak and strong points. Due to limited hospital funding for lab analyses, more lab investigations could not be added. One of the significant limitations was convenience sampling. All recruited samples were analyzed in one authorized lab to avoid variations leading to misidentification.

## 5. Conclusions

The findings conclude that lipid levels deranged independently regardless of type 2 diabetes mellitus and present as an early onset in type 2 diabetes instead of later stage complication. These derangements of lipid levels are an independent risk factor for future cardiovascular pathology.

In this study, HDL-C and TGs were observed to be the most desired predictors of dyslipidemia. The presence of deranged lipid profiles in individuals with (in newly diagnosed diabetics) or without hyperglycemic status seems to be concerning in the public health sector. This shows an independent risk factor for future cardiovascular events. The findings suggest that real-time solution management strategies are needed to control the disease outcome and related complications.

## Figures and Tables

**Table 1:** Independent Samples *t*-test to compare the mean difference in data variables (*p*-value ≤ 0.05).

Variables	Group I (Diabetic Dyslipidemia)	Group II (Dyslipidemia)	*p*-Value
Age (years)	52.13 ± 10.75	47.33 ± 12.79	0.002 *
BMI (kg/m^2)^	26.22 ± 4.53	26.76 ± 6.20	0.440
FBS (mmol/L)	11.54 ± 4.92	5.55 ± 0.47	0.000 *
HDL-C (mmol/L)	0.79 ± 0.25	0.87 ± 0.24	0.035 *
LDL-C (mmol/L)	2.96 ± 1.02	2.82 ± 0.96	0.287
TC (mmol/L)	5.09 ± 1.19	4.54 ± 1.33	0.001 *
Triglycerides (mmol/L)	3.25 ± 1.99	2.41 ± 1.24	0.000 *
HbA1c (mmol/L)	8.78 ± 2.14	5.38 ± 0.64	0.000 *

BMI: body mass index, FBS fasting blood sugar, HDL-C: high-density lipoprotein cholesterol, LDL-C: low-density lipoprotein cholesterol, TC: total cholesterol; Data presented as Mean ± SD; * p-value < 0.05 as statistically significant.

**Table 2:** Linear Regression Analysis for Predictors of Dyslipidemia.

Parameter	R^2^	Gender Male = 0, Female = 1	Age	Socio−Economic Status	Family History	Physical Activity	BMI
HDL	0.034	0.030 (−0.039, 0.099)	−0.002 (−0.004, 0.001)	0.018 (−0.018, 0.054)	−0.005 (−0.017, 0.007)	−0.043 (−0.80, −0.006)	0.000 (−0.042, 0.041)
*p*-value		0.391	0.280	0.330	0.395	0.022 *	0.994
LDL	0.021	0.107 (−0.177, 0.391)	−0.004 (−0.015, 0.007)	−0.067 (−0.216, 0.082)	−0.024 (−0.073, 0.025)	−0.043 (−0.194, 0.109)	−0.090 (−0.260, 0.081)
*p*-value		0.449	0.492	0.377	0.334	0.578	0.302
TGs	0.039	−0.633 (−1.135, −0.132)	−0.001 (−0.021, 0.09)	0.120 (−0.143, 0.383)	−0.014 (−0.100, 0.072)	0.163 (−0.104, 0.431)	0.058 (−0.244, 0.359)
*p*-value		0.014 *	0.889	0.369	0.751	0.230	0.706
TC	0.040	0.334 (−0.021, 0.689)	0.008 (−0.006, 0.022)	−0.064 (−0.050, 0.122)	−0.062 (−0.123, −0.001)	−0.042 (−0.231, 0.148)	−0.135 (−0.348, 0.079)
*p*-value		0.065	0.275	0.496	0.045 *	0.665	0.215

HDL-C: high-density lipoprotein cholesterol, LDL-C: low-density lipoprotein cholesterol, TGs: triglycerides, TC: total cholesterol; Data presented as B (unstandardized coefficient), 95% Confidence Interval; * *p*-value (<0.05) as statistically significant.

## Data Availability

Not Applicable.
